# Gene Flow Between Populations With Highly Divergent Mitogenomes in the Australian Stingless Bee, *Tetragonula hockingsi*

**DOI:** 10.1002/ece3.70475

**Published:** 2024-11-13

**Authors:** Genevieve Law, Carmen R. B. da Silva, Inez Vlasich‐Brennan, Benjamin A. Taylor, Brock A. Harpur, Tim Heard, Scott Nacko, Markus Riegler, James B. Dorey, Mark I. Stevens, Nathan Lo, Rosalyn Gloag

**Affiliations:** ^1^ School of Life and Environmental Sciences University of Sydney Sydney New South Wales Australia; ^2^ School of Biological Sciences Monash University Melbourne Victoria Australia; ^3^ School of Natural Sciences Macquarie University Sydney New South Wales Australia; ^4^ Department of Entomology Purdue University West Lafayette Indiana USA; ^5^ Hawkesbury Institute for the Environment Western Sydney University Penrith New South Wales Australia; ^6^ School of Earth, Atmospheric, and Life Sciences University of Wollongong Wollongong New South Wales Australia; ^7^ Earth & Biological Sciences South Australian Museum Adelaide South Australia Australia; ^8^ School of Biological Sciences University of Adelaide Adelaide South Australia Australia

**Keywords:** hybridization, Mito‐nuclear coevolution, speciation, stingless bee

## Abstract

Coadaptation of mitochondrial and nuclear genes is essential for proper cellular function. When populations become isolated, theory predicts that they should maintain mito‐nuclear coadaptation in each population, even as they diverge in genotype. Mito‐nuclear incompatibilities may therefore arise when individuals from populations with divergent co‐evolved mito‐nuclear gene sets are re‐united and hybridise, contributing to selection against inter‐population hybrids and, potentially, to speciation. Here, we explored genetic divergence and gene flow between populations of a stingless bee (*Tetragonula hockingsi*) that have highly divergent mitogenomes. We identified three distinct populations across the species' 2500 km range on the east coast of Queensland (Australia): ‘Cape York’, ‘Northern’, and ‘Southern’. The mitogenomes of each population showed > 12% pairwise nucleotide divergence from each other, and > 7% pairwise amino acid divergence. Based on nuclear SNPs from reduced representation sequencing, we identified at least two zones of gene flow between populations: a narrow natural zone between Northern and Southern populations (coinciding with a biogeographic barrier, the Burdekin Gap), and an artificial zone at the southern edge of the species' distribution, where Cape York, Northern, and Southern mito‐lineages have been brought together in recent decades due to beekeeping. In the artificial hybrid zone, we also confirmed that males of all three mito‐lineages were attracted to the mating aggregations of Southern queens, consistent with inter‐population hybridisation. Populations of *T. hockingsi* thus appear to be in the ‘grey zone’ of the speciation continuum, having strong genetic differentiation but incomplete reproductive isolation. Among the nuclear SNPs most differentiated between Northern and Southern populations, several were associated with genes involved in mitochondrial function, consistent with populations having co‐diverged mito‐nuclear gene sets. Our observations suggest that coadapted sets of mitochondrial and nuclear genes unique to each population of *T. hockingsi* may play a role in maintaining population boundaries, though more study is needed to confirm the fitness costs of mito‐nuclear incompatibilities in hybrid individuals.

## Graphical abstract

We investigate genetic divergence and gene flow between populations of a stingless bee (*Tetragonula hockingsi*) that have highly divergent mitogenomes. We identify at least two zones of gene flow between populations: a narrow natural zone coinciding with a biogeographic barrier, and an artificial zone where mito‐lineages have been brought together in recent decades due to beekeeping. Populations of *T. hockingsi* thus appear to be in the ‘grey zone’ of the speciation continuum, having strong genetic differentiation but not reproductive isolation.

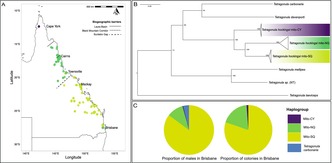

## 1 Introduction

The mitochondrial genome (mitogenome) has long held a central role in identifying species and delineating species boundaries (Hill 2016; Linares et al. 2009). In animals, mitochondrial genes accumulate substitutions at a faster per‐nucleotide rate than nuclear genes, typically show uniparental (maternal) inheritance, and rarely undergo recombination (Ladoukakis and Zouros 2017), making them popular species barcode genes (Hill 2016; Song et al. 2018). A variety of processes can lead to patterns of mitochondrial divergence that fail to reflect broader species divergence, such as mitogenome introgression between populations, positive selection on mitogenomes, or endosymbionts (Despres 2019; Hinojosa et al. 2019; Linares et al. 2009). Nevertheless, high divergence at mitochondrial genes is often the first line of genetic evidence used to identify taxa as separate species, particularly for taxa where morphologically cryptic species are common (Cairns et al. 2021; da Silva et al. 2011; Hill 2016; Linares et al. 2009; Martínez et al. 2023; Song et al. 2018).

In addition to being effective markers of population divergence, mitochondrial genes (mt genes) can also become a barrier to gene flow through genetic incompatibility of hybrids between diverged populations and therefore create species boundaries (Burton and Barreto 2012; Hill 2016). Incompatibilities between two or more interacting genes, also known as Bateson‐Dobzhansky‐Muller incompatibilities (BDMIs), are thought to be key drivers of speciation (Dobzhansky 1982; Unckless and Orr 2009). Although BDMIs were originally envisaged to be incompatibilities between different nuclear genes, such incompatibilities can also arise from interactions between mt genes and those nuclear genes whose products interact with mt genes and their products (N‐mt genes). Indeed, these mito‐nuclear BDMIs might be particularly deleterious because they affect respiration and other critical mitochondrial functions (Havird and Sloan 2016; Lechuga‐Vieco, Justo‐Méndez, and Enríquez 2021). In this scenario, mito‐nuclear incompatibilities develop when two populations become isolated by geographic or ecological barriers (Tobler, Barts, and Greenway 2019). On secondary contact, inter‐population hybrids then carry mitogenomes that must interact with some N‐mt genes of a foreign nuclear environment; that is, N‐mt genes with which they have not coevolved (Burton and Barreto 2012). Given that mitogenomes tend to diverge rapidly, mito‐nuclear incompatibilities have been proposed to be among the first incompatibilities to arise between diverging populations (Hill 2017). While fitness costs to hybrids from mito‐nuclear incompatibilities have been documented (Ellison and Burton 2008; Ellison, Niehuis, and Gadau 2008; Pereira et al. 2021; Zhang, Montooth, and Calvi 2017), the prevalence of these BDMIs in natural populations is unclear (Burton 2022). Studies across diverse taxa with populations at different stages of divergence are thus needed to better understand the role of mito‐nuclear coevolution in the speciation process.

Emerging evidence suggests that the Australian stingless bee *Tetragonula hockingsi* (Cockerell 1929) (Apidae: Meliponini) may be an ideal system in which to study mito‐nuclear speciation. Previous evidence from nuclear loci (microsatellites) and one mitochondrial gene (*COI*) suggests that *T. hockingsi* can be divided into at least two populations across its range on the north‐east coast of Queensland, Australia (Brito et al. 2014; Franck et al. 2004): a Northern population defined by one *COI* haplogroup (which we call ‘mito‐NQ’), and a Southern population, defined by another *COI* haplogroup (which we call ‘mito‐SQ’). The mito‐NQ and mito‐SQ populations appear to be separated by a 200 km stretch of dry savannah known as the Burdekin Gap, which is a barrier to gene flow for a range of closed‐forest taxa (Bryant and Krosch 2016) and which is likely to be poor habitat for stingless bees.

Mitochondrial divergence between the Northern and Southern *T. hockingsi* populations is high, with the mito‐NQ and mito‐SQ *COI* haplogroups showing 10.4% pairwise genetic distance (Brito et al. 2014; Françoso et al. 2019). *Tetragonula* stingless bees have an atypical mitogenome structure characterised by long inverted repeats, LIRs (Françoso et al. 2023; Li et al. 2024). In the case of *T. hockingsi*, the presence of LIRs has resulted in a 30,662 kb mitogenome molecule with two identical copies of all genes and most tRNAs (Françoso et al. 2023). If and how this mitogenome structure affects mitochondrial substitution rates remains unknown, but there is some evidence that *Tetragonula* mitogenomes diverge quickly, relative to those of other meliponine bees (Françoso et al. 2019, 2023). Regardless of the underlying mechanistic cause, the high divergence of *COI* between *T. hockingsi* populations suggests they may be strong candidates for showing mito‐nuclear incompatibilities on secondary contact.

Furthermore, *T. hockingsi* are ideal species in which to examine mito‐nuclear incompatibilities due to their popularity among beekeepers, and consequential movement across large regions. *Tetragonula hockingsi* are readily kept in wooden nest boxes, and increasingly used as an agricultural pollinator or traded by hobbyists (Halcroft et al. 2013). The rising popularity of stingless beekeeping in Australia has led to the transportation of colonies, often across large distances (Byatt et al. 2016; Chapman et al. 2017). In a previous study, a number of ‘misplaced’ mito‐NQ colonies were detected among the natural nests of a forest fragment in Brisbane, a city at the southernmost edge of the species range (Xia 2022). As Brisbane has an active stingless beekeeping community, it seems likely that these Northern‐lineage individuals in the south are the result of hive movements. If so, the presence of mito‐NQ individuals in Brisbane provides a further opportunity to examine the potential for hybridisation between *T. hockingsi*'s divergent populations, and also to better understand the implications of hive movements on the species' population structure and health.

**FIGURE 1:**
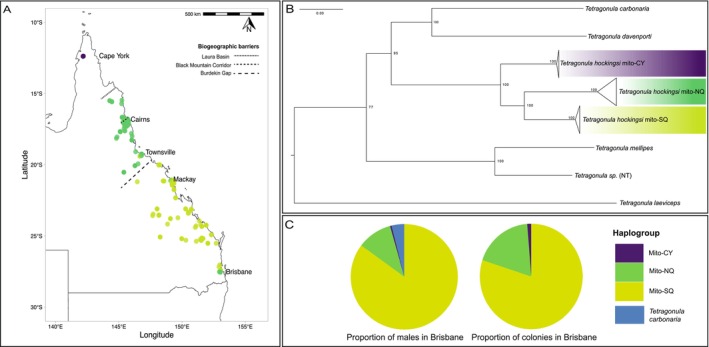
(A) Locations of 865 *Tetragonula hockingsi* colonies samples from across their range, coloured by haplogroup: Mito‐CY (purple), mito‐NQ (dark green), mito‐SQ (yellow‐green). (B) ML phylogeny of concatenated mt amino acid sequences, inferred using IQ‐TREE. Collapsed lineages of *T. hockingsi* haplogroups each contain two samples. The scale represents substitutions per site. Node support values are bootstrap values calculated with UFBoot2 in IQ‐TREE. (C) Pie charts representing total proportions of males and colonies of each haplogroup sampled in Brisbane, respectively (including heterospecific males: Blue).

Here, we aimed to establish whether *T. hockingsi* populations with divergent mitogenomes were reproductively isolated, and whether population divergence was consistent with mito‐nuclear coevolution. To do this, we first examined *COI* sequences and whole mitogenomes from across *T. hockingsi*'s range to map the distribution of each mt‐haplogroup and to determine the extent of mitogenome divergence between the populations. Our sampling covered the two known populations (mito‐NQ and mito‐SQ) and identified a third population, based on another divergent haplogroup (mito‐CY), from north‐western Cape York (Figure 1A). We then assess the prevalence of mito‐NQ colonies in Brisbane (at the Southern range edge), and determine whether males of all haplotypes at this location join the mating aggregations of mito‐SQ queens, to assess whether pre‐zygotic reproductive barriers exist between the populations. We then analysed population structure in genome‐wide nuclear SNPs generated from reduced‐representation sequencing of *T. hockingsi* from across their range to examine nuclear divergence and hybridization between the populations. Finally, we looked for evidence that nuclear loci that were strongly diverged between populations were associated with mitochondrial function, consistent with mito‐nuclear coevolution and the possibility that mito‐nuclear interactions could be a source of hybrid incompatibility.

## 2 Materials and Methods

### 2.1 Distribution and Diversity of Mt‐Haplogroups

To better map the geographic distribution of mt‐haplogroups across *T. hockingsi*'s full geographic range, we sequenced a fragment of *COI* from 865 *T. hockingsi* workers, sampled from hives (provided by beekeepers; *N* = 318), natural nests (*N* = 72), by sweep‐netting flowers (*N* = 470), and from swarms and male roosts (*N* = 6); Figure 1A, Table S1. In all cases, samples were collected directly onto 100% ethanol. We extracted DNA using 5% Chelex w/v in 1:10 TE buffer (Walsh, Metzger, and Higuchi 2013).

We then amplified an approximately 500 bp fragment of the *COI* gene using primers from Françoso et al. (2019) (BarhockF and BarhockR; Table S2). These primers amplify *T. hockingsi COI* sequences without amplifying the nuclear pseudogenes (NUMTs) that are known to occur in this species (Françoso et al. 2019) and can be used to effectively identify the mito‐SQ and mito‐NQ haplotypes of *T. hockingsi* (Paul et al. 2023). Our PCR protocol used 20 μL reactions, each containing 2 μL DNA template, 0.2 μL of 5 U/μL Taq‐Ti polymerase, 0.4 μL each of 20 μM BarhockF and BarhockR, and 2 μL 10× PCR buffer. We ran PCR with 35 cycles and an annealing temperature of 50°C; PCR products were then sent to Macrogen Inc. (Seoul, Republic of Korea) for Sanger sequencing. For a subset of the total samples (*n* = 18), *COI* sequencing was instead performed using single‐molecule real‐time sequencing (SMRT) (Hebert et al. 2018) in the PacBio Sequel platform (Pacific Biosciences, Menlo Park, CA, USA) at the Canadian Centre for DNA Barcoding (CCDB), in the University of Guelph, Ontario, Canada.

We trimmed and aligned sequences in Geneious Prime version 2022.1 (Kearse et al. 2012); we removed from the dataset any samples with close sequence matches to the morphologically similar *T. carbonaria* (Paul et al. 2023). From the remaining alignment (160 of 189 total), we determined that there were three major mt‐haplogroups (mito‐NQ, mito‐SQ, and mito‐CY): *COI* sequences were initially compared within and between groups by calculating overall mean distance and between group mean distance (expressed as percentages) using MEGA version 11.0.11 (Tamura, Stecher, and Kumar 2021) with the p‐distance model. *COI* haplotypes within a haplogroup were very similar at the nucleotide level (0%–1%), while they differed by 12%–14% between groups.

To further assess the extent of divergence in mt‐haplogroups, we assembled whole mitogenomes for six *T. hockingsi* individuals (two mito‐NQ, two mito‐SQ, and two mito‐CY; Table S1). We extracted DNA using a Qiagen DNeasy Blood & Tissue Kit (Cat No./ID: 69506; Qiagen, Hilden, Germany). Sequencing of mito‐SQ and mito‐NQ samples was performed at the Australian Genome Research Facility (AGRF) using Illumina HiSeqX, and sequencing of the mito‐CY mitogenomes was performed by Novogene using the Illumina NovaSeq PE150. We mapped reads to a *T. hockingsi* reference mitogenome (OQ918629; Françoso et al. 2023) using Geneious Prime; mean coverage ± standard deviation (s.d.) was 3162 reads ± 603 reads. We then calculated between‐group genetic distance separately for all nucleotides, all tRNA and rRNAs (concatenated), and for amino acid sequences (concatenated for all 13 mt‐genes) again using MEGA (p‐distance model).

Finally, to compare the divergence of mt‐haplogroups within *T. hockingsi* to those between recognised species of *Tetragonula*, we constructed a maximum likelihood phylogeny of concatenated amino acid sequences using the IQ‐TREE webserver (Nguyen et al. 2014; Trifinopoulos et al. 2016). This phylogeny included our six *T. hockingsi* samples plus one *T. carbonaria* mitogenome (Smith 1854) (GenBank accession number OQ918628.1; Françoso et al. 2023), and four species assembled by Li et al. (2024): *T. davenporti* (Franck et al. 2004) (GenBase: C_AA057847.1), *T. laeviceps* (Smith 1857) (C_AA057808.1), *T. mellipes* (Friese 1898) (C_AA057849.1), and *Tetragonula* sp. “NT” (C_AA057850.1). The latter is a currently unnamed *Tetragonula* species from the Northern Territory of Australia (Hereward J., pers. comm.). Concatenated amino acid sequences were partitioned to allow for different substitution models (Table S3), which were identified using IQ‐TREE's ModelFinder (Kalyaanamoorthy et al. 2017). Ultrafast bootstrap values were identified using IQ‐TREE's UFBoot2 (Hoang et al. 2017) using default settings. The tree was then reformatted in FigTree (http://tree.bio.ed.ac.uk/software/figtree/) and R version 4.2.0 (R Core Development Team 2022) and RStudio version 2022.02.3 using ape version 5.8 (Paradis and Schliep 2018).

### 2.2 Prevalence of Northern Haplotypes in the Southern Region

Previous evidence (Xia 2022) indicated there were some natural colonies with mito‐NQ haplotypes occurring in a localised region of Brisbane, a city in the southern‐most part of the *T. hockingsi* range (Figure 1A). In addition to previously collected *T. hockingsi*, we performed more intensive sampling of *T. hockingsi* workers and males from Brisbane between 2019 and 2022 to (i) determine the frequency of northern (mito‐NQ or mito‐CY) haplotypes in this southern region and (ii) determine whether any detected mito‐NQ or mito‐CY males were attracted to virgin queens of the local (mito‐SQ) haplotype. We collected workers from natural colonies (*N* = 14 colonies), ‘rescued colonies’ (in other words, those recently relocated into hives from other cavities, such as water meter boxes or felled trees; *N* = 153 hives), and flowers (*N* = 16 areas, where each area was approximately 0.25 km^2^; Table S1).

We collected males from 15 mating aggregations (male swarms and roosts) that formed adjacent to re‐queening colonies. Male *Tetragonula* fly on average 2–3 km (though up to 20 km) from their natal nests to join mating aggregations, and aggregations typically contain hundreds of males from dozens of different colonies, thereby providing a valuable snapshot of haplotype diversity in a local area (Bueno et al. 2022). Nine naturally occurring aggregations were opportunistically sampled, while six aggregations were encouraged to form at hived colonies by ‘splitting’, a hive propagation technique in which one half of a hived nest is placed into a new box, stimulating a virgin queen to mate and thus attracting males (Bueno et al. 2022; Paul et al. 2023; Table S1). We also sampled workers from the colonies that attracted the aggregations (in other words, the closest colony and/or recently split hive) to establish the mt‐haplotype of the virgin queens. We used molecular and morphological techniques to confirm that individuals collected at male aggregations were indeed males, and not swarming workers (see method in Supporting Information).

In total, we determined the mt‐haplogroup for 213 workers (1 per colony, 1–2 per location for samples from flowers) and 813 males (20–50 per aggregation; mean ± s.d. = 47 males ± 19.76) from Brisbane, via *COI* sequencing using the same protocols as described above. We used a Fisher's Exact Test to compare the proportions of mito‐NQ and mito‐SQ in our total dataset of males versus workers using the package *stats* in RStudio. We assumed that equal proportions would indicate that mito‐NQ males were represented at mating aggregations at rates similar to their local abundance (in other words, they had no aversion to mito‐SQ queens).

To assess whether we could identify more precisely the origin of mito‐NQ and mito‐CY lineages now in Brisbane, we also inferred a maximum likelihood phylogenetic tree of *COI* sequences using IQ‐TREE (Nguyen et al. 2014). After removing redundant sequences, the tree was generated from an alignment of sequences of mito‐NQ and mito‐CY samples identified in Brisbane (*N* = 22), as well as 78 other *T. hockingsi* from locations spanning their distribution, and *T. carbonaria* (*N* = 3) as the outgroup (Table S1). Identical nucleotide sequences were identified using Geneious and only one sample per unique sequence was retained (167 sequences were removed). Ultrafast bootstrap values were added with default settings using UFBoot2 and an appropriate substitution model was identified using ModelFinder: the most appropriate model was TN + F + G4. The tree was then reformatted using ape.

### 2.3 Population Structure Based on Nuclear SNPs

**FIGURE 2:**
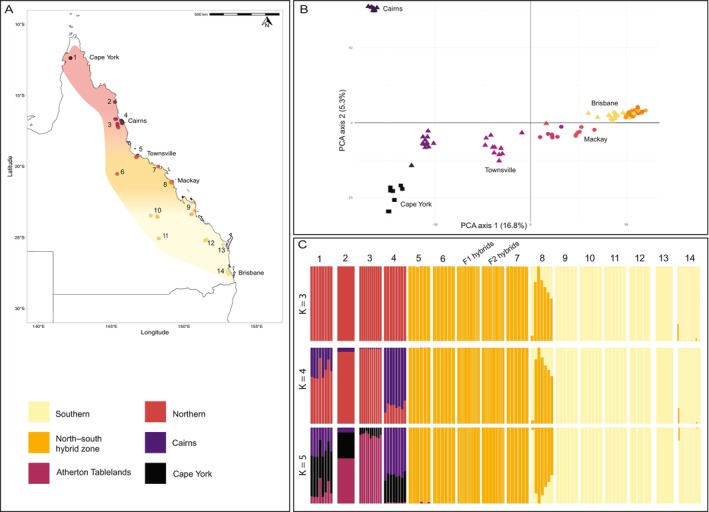
(A) Sampling locations of all *Tetragonula hockingsi* samples used for DArTseq (*N* = 90); sample locations are gradient coloured according to subpopulations (from yellow in the south to purple in the north; as also in Figure 2B), while *T. hockingsi*'s background distribution is coloured according to the populations indicated by DAPC analysis (see legend; and also Figure 2C, *K* = 3). Subpopulations are also numbered according to those used in the DAPC analysis (Figure 2C). (B) PCA plot of *T. hockingsi* DArTseq samples, with samples coloured according to subpopulation and shapes referring to the mt‐haplogroup of the sample (square = mito‐CY, triangle = mito‐NQ, and circle = mito‐SQ); PCA axis 1 explained 16.8% of variation while PCA axis 2 explained 5.3% of variation (C). DAPC analysis of *T. hockingsi* DArTseq samples at *K* = 3 (top panel), *K* = 4 (middle panel) and *K* = 5 (bottom panel). Subpopulations are separated and numbered (corresponding to the map in Figure 2A), while F1 and F2 simulated hybrids (simulations of hybridisation between Northern and Southern populations) are shown in the middle of each plot.

We extracted genomic DNA from 94 *Tetragonula* spp. workers using a Qiagen DNeasy Blood & Tissue Kit. These samples included 90 *T. hockingsi* that spanned the species' distribution (Figure 2A) and four samples from outgroup species (*T. carbonaria*, *T. sapiens*, and two *T. clypearis*). Among the *T. hockingsi* samples, we included 10 samples that were collected in the Southern population but which had a Northern mt‐haplotype (in other words, mito‐NQ; these samples were mostly from Brisbane, *N* = 8, though one was from just south of the Burdekin Gap); the clustering pattern of these samples was used to indicate whether or not they hybridise with the local populations. We also included one mito‐SQ sample provided by a beekeeper in Townsville, just north of the Burdekin Gap (a location where all other samples were mito‐NQ).

DArTseq reduced‐genome sequencing was performed by Diversity Arrays Technology (DArT) Pty Ltd. (Canberra, ACT, Australia). This sequencing approach is a variant of RAD‐seq in which combinations of restriction enzymes target regions likely to harbour informative SNPs because they are low‐copy, hypo‐methylated (regions that therefore have increased transcription), polymorphic, and/or gene‐rich regions (Bird 1986; Kilian et al. 2012; van Deventer et al. 2020). Reduced representation libraries from this DNA were then generated at DArT following a digestion/ligation process (Kilian et al. 2012) with two restriction enzyme adaptors (*PstI* and *MseI*), where the *PstI* compatible adaptor has a flow cell attachment sequence (Illumina, San Diego, CA, USA), sequencing primer sequence and barcodes for sample identification (Elshire et al. 2011). Only fragments that contained both adaptors were amplified via PCR (Kilian et al. 2012). After PCR, equimolar amounts of each sample were pooled, prior to sequencing on Illumina HiSeq2500 (single‐read, 77 cycles, 1.25 M reads per sample). Reads were then processed for SNP identification using DArT's proprietary analytical pipelines to remove low quality sequences and those with poor repeatability. This initial pipeline returned 52,401 SNPs which we then further filtered to retain only the highest quality markers, using dartR version 2.9.7 (Mijangos et al. 2022). We removed loci that were monomorphic, had missing values (call rates < 95%), minimal read depth (read depth < 10), low reproducibility (RepAvg < 95%), and low minor allele frequencies (MAF < 0.02). Where multiple loci were found on the same sequencing fragment, we selected one locus at random to retain. In all, 8353 SNPs remained after filtering and were used in subsequent analyses.

To assess how population structure in nuclear genomes corresponded to that of mitochondrial genomes, and specifically to determine whether mito‐NQ and mito‐SQ individuals were interbreeding, we performed five different analyses. First, we conducted a principal component analysis (PCA) using gl.pcoa in dartR to qualitatively examine the differences between haplogroup‐defined populations (Cape York, Northern, and Southern) and regions within populations (14 total regions, Figure 2A–C: Cape York, Cooktown, Atherton Tablelands, Cairns, Townsville, Pentland, Bowen, Mackay, Greater Rockhampton, Maryborough, Central Highlands, Carnarvon Gorge, Mount Perry, and Brisbane).

Second, we calculated pairwise *F*_*ST*_ values between populations, and between regions within populations, using stamppFst in StAMPP version 1.6.3 (Pembleton, Cogan, and Forster 2013) with the Weir and Cockerham method (Weir and Cockerham 1984), where larger *F*_*ST*_ values indicate greater genetic differentiation between populations. The subpopulation locations were almost the same as above; however, Cooktown and Maryborough were excluded because only one individual was collected from each of these locations.

Third, we calculated an analysis of molecular variance (AMOVA) test (Excoffier, Smouse, and Quattro 1992) using the R packages poppr version 2.9.6 (Kamvar, Tabima, and Grünwald 2014) and ade4 version 1.7 (Dray and Dufour 2007). This test was used to determine the significance of contributions to genetic variance according to the following hierarchy: Cape York, Northern, and Southern were compared at the population level, and regions were used at the subpopulation level as defined for *F*_*ST*_ calculations.

Fourth, we conducted a discriminant analysis of principal components (DAPC) (Jombart, Devillard, and Balloux 2010), using the function dapc in the package adegenet version 2.1.10 (Jombart 2008). Compared to traditional structure‐based methods such as STRUCTURE (Pritchard, Stephens, and Donnelly 2000), DAPC is less computationally intensive and does not involve assumptions of genetic processes like Hardy–Weinberg equilibrium (Jombart, Devillard, and Balloux 2010; Miller, Cullingham, and Peery 2020). DAPC uses PCA to determine clusters within the dataset based on principal components, which are then refined by the main discriminant analysis. We selected the number of clusters retained (K) based on Bayesian Information Criterion (BIC) values generated by DAPC, where we considered lower BIC values to better describe population structure. We aimed to avoid overfitting of the DAPC model by finding the number of principal components that corresponded to the lowest α score (calculated by adegenet); in this case one principal component. We also included simulated north–south hybrids in our DAPC in order to investigate the likelihood of gene flow occurring between these populations. Hybrids were simulated using adegenet, with a ‘Northern parent’ dataset comprised of all individuals from the Atherton Tablelands, Townsville (except the one mito‐SQ individual), Pentland, and Bowen, and a ‘Southern parent’ dataset comprised of Mackay (except the one mito‐NQ individual), Rockhampton, Central Highlands, Carnarvon Gorge, and Maryborough. We first simulated 20 F1 hybrids, which were then used as the parent population to simulate 20 F2 hybrids. We considered that real samples in our dataset were likely to represent actual recent hybrids (in other words, evidence of current gene flow) if they clustered together with these simulated hybrids in genetic space in DAPC analyses (Stronen et al. 2022).

Finally, we created a maximum likelihood phylogenetic tree using IQ‐TREE to visualise the relationship between samples based on nuclear SNPs and to look for evidence of discordance with mt‐haplotypes. The best supported substitution model was TPM3u + F + G4 (BIC = 305097.14). The analysis was conducted on concatenated base pairs representing each SNP, with heterozygous SNPs encoded using IUPAC ambiguity codes (Melville et al. 2017).

### 2.4 Nuclear Loci That Contribute to Population Divergence

Given the high divergence of mitogenomes between *T. hockingsi* populations, we predicted that N‐mt genes (those nuclear genes whose products interact with mitochondrial gene products) would be among the genes most highly diverged between populations, due to mito‐nuclear coevolution (Morales et al. 2018; Storz 2005). To test this prediction, we focused on a comparison of the Northern and Southern populations (sample sizes were insufficient for the Cape York population). We identified outlier loci in the filtered DArT dataset using two methods, where outlier loci were defined as those that were most strongly differentiated between the mito‐NQ and mito‐SQ populations.

First, we identified loci with the top 1% of *F*_*ST*_ values that distinguished between the Northern and Southern populations using the function diffCalc in the R package diveRsity version 1.9.90 (Keenan et al. 2013). To select this set of loci we used two comparisons: we compared the ‘broad range’ Northern and Southern populations, as defined by their haplotypes, but excluding putative hybrids (in other words, all individuals from Brisbane and two individuals with ‘misplaced’ mt‐haplotypes from the Burdekin Gap region) and excluding individuals from Cairns (which PCA indicated to be divergent from elsewhere in the north), and we compared the ‘narrow range’ Northern and Southern populations (in other words, samples from the northern (Pentland and Townsville) and southern (Bowen and Mackay) ends of the Burdekin Gap hybrid zone). We then retained only the loci that were identified by both comparisons. This approach therefore did not consider every locus diverged between the populations, but rather gave us a conservative set of the most highly diverged loci in our SNP dataset that might have been driven by co‐evolution with mitochondrial genomes.

Second, we ran a DAPC analysis (*K* = 2, PC = 1 after α optimisation) using the function dapc in adegenet to identify outlier loci between the Northern and Southern populations (again excluding individuals that could not be unambiguously assigned to one or the other population, as described above). To do this, we used the function snpzip (in adegenet) to identify SNPs that contribute to the population structure identified by the DAPC analysis using the clustering method ‘average’.

The sequence probes provided by DArT on which the outlier SNPs were found were then mapped to a reference *T. carbonaria* nuclear genome (Taylor et al., *unpubl*.) in Geneious (‘medium sensitivity and fast’, up to 25 iterations). We identified annotated genes that were located close to (and thus likely to be linked with) the outlier loci. The rate of recombination and the level of linkage disequilibrium influence the distance at which genes and nearby SNPs will be inherited together, and thus the extent to which patterns of SNP allele fixation reflect fixation of nearby genes through genetic hitchhiking (Storz 2005). Eusocial hymenopterans are known to have high recombination rates and thus linkage disequilibrium decays steeply with distance (Ross et al. 2015). In honey bees (*Apis mellifera*) (Linnaeus 1758), linkage disequilibrium has been estimated to decay between 1500 bp (Wallberg et al. 2014) and 10,000 bp (Whitfield et al. 2006). However, the recombination rate in the stingless bee *Frieseomelitta varia* (Lepeletier 1836) was estimated as lower than that of honey bees: 12.6 cM/Mb (Waiker et al. 2021) versus 19.0–26.0 cM/Mb in *A. mellifera* (Beye et al. 2006; Wallberg, Glémin, and Webster 2015). For our analysis, we designated genes within 5000 bp of an outlier SNP were likely to be linked. For genes associated with outlier loci, we assessed whether they were likely to localise in the mitochondria using MULocDeep version 1.7 (Jiang et al. 2021), which analyses protein structure to predict localisation of proteins within the cell without reliance on the detection of target signals that are variable in mitochondrially localised proteins (Habib, Neupert, and Rapaport 2007; Jiang et al. 2021; Nithya 2019). We also searched the GenBank database using BLASTn (https://blast.ncbi.nlm.nih.gov/Blast.cgi) to determine if genes had known homologues. All genes that were identified with E values of less than or equal to 1 × 10^−5^ were recorded (Montero‐Mendieta et al. 2019). We then determined whether identified genes produced proteins that interacted with the mitochondria by searching their gene ontology (GO) terms for those related to the mitochondria using UniProt (The Uniprot Consortium 2018).

## 3 Results

### 3.1 Distribution and Divergence of t‐Haplogroups

*COI* haplotypes revealed a clear structuring of *T. hockingsi* (*n* = 868) into three populations (Figure 1A). There was no gradual cline in mt haplotype frequencies evident from the locations we sampled; rather, there was an abrupt switch from the mito‐NQ to mito‐SQ haplotypes somewhere within the ~200 km region between Townsville and Bowen (just one mito‐SQ individual was sampled immediately north of this zone, and one mito‐NQ immediately south of it, both from hived colonies kept by beekeepers). Likewise, no mito‐CY individuals were detected at the edge of the mito‐NQ population, although the unsampled region between these populations was large (approximately 500 km).

Based on whole mitogenomes, the three mt‐haplogroups showed 11.63 ± 0.41% uncorrected pairwise nucleotide divergence from each other. This included sequence polymorphisms across all tRNAs and rRNAs (mito‐CY vs. mito‐SQ = 5.22%; mito‐NQ vs. mito‐SQ = 4.44%; mito‐NQ vs. mito‐CY = 5.48%) and non‐synonymous divergence across all 13 protein‐coding genes (mito‐CY vs. mito‐SQ = 8.88%; mito‐NQ vs. mito‐SQ = 7.69%; mito‐NQ vs. mito‐CY = 7.53%; Figure 1B **)**. In contrast, within‐haplogroup samples had identical or near identical amino acid sequences (*n* = 2 for each group; mito‐NQ = 0.00%, mito‐CY = 0.00%, mito‐SQ = 1.00%), as did all individuals within‐haplogroup at *COI* (mito‐NQ = 0.00%, *n* = 44; mito‐CY = 0.01%, *n* = 19; Mito‐S = 0.00%, *n* = 37; Figure S1).

### 3.2 Prevalence of Northern Haplotypes in the South

*Tetragonula hockingsi* workers (colonies) and males carrying northern (mito‐NQ or mito‐CY) mt‐haplotypes comprised approximately one sixth of all samples collected in Brisbane, a city close to the species' southern range edge (Figure 1C). The majority of these ‘misplaced’ haplotypes were mito‐NQ (18% of all colonies, 28/128 workers, and 11% of all males; 74/672 males), with a small proportion being mito‐CY (1% of colonies, 2/128 workers, and 0.45% males, 3/672 males); Figures S2, S3. The remaining *T. hockingsi* in Brisbane were mito‐SQ, and thus the expected haplotype given their southern location.

Males with mito‐NQ and mito‐CY haplotypes were confirmed to join mating aggregations outside colonies with mito‐SQ virgin queens, with 12 of the 15 mating aggregations having at least one northern haplotype male. Moreover, the proportion of mito‐NQ and mito‐SQ males in mating aggregations was statistically no different from the population of colonies (workers) sampled in Brisbane (Fisher's exact test; *p* = 1.00, 95% confidence interval = 0.025) suggesting that males do not discriminate against mito‐SQ queens when choosing mating aggregations. Some mating aggregations also included males of another species, *T. carbonaria* (*N* = 8 aggregations; 6.60% ± 5.77% per aggregation). Based on sequence similarity at *COI*, almost all Brisbane mito‐NQ workers clustered together, suggesting a possible single origin for these mito‐NQ lineages (Figure S1). However, the very low divergence within *COI* haplogroups makes a conclusive assignment of origin difficult.

### 3.3 Population Structure Based on Nuclear SNPs

Nuclear genome SNPs revealed geographic structuring that was broadly consistent with the three haplotype‐defined populations (mito‐NQ, mito‐SQ, and mito‐CY), as indicated by our PCA (Figure 2B) and phylogenetic analyses (Figure S4). We found further support for population structuring through *F*_*ST*_ and AMOVA analyses (Table 1). More specifically, *F*_*ST*_ values between the three populations were moderately high (Northern and Cape York = 0.108, Northern and Southern = 0.145, Cape York and Southern = 0.283), and there was also significant variance between subpopulations (Table 1; Figure S5).

**TABLE 1:** Contributions to genetic variance in *Tetragonula hockingsi* according to an AMOVA test with the following hierarchy: Cape, Northern and Southern populations; Cape York, Atherton Tablelands, Cairns, Townsville, Pentland, Bowen, Mackay, Greater Rockhampton, Central Highlands, Carnarvon Gorge, Mount Perry, and Brisbane regions (subpopulations). Individuals from Cooktown and Maryborough were not included in this analysis due to low sample sizes.

Source of variance	φ statistic	Variance components	% variation	*p*
Between populations	0.138	305.118	13.830	0.01
Between regions (within populations)	0.106	200.955	9.109	0.01
Between samples (within regions)	0.064	108.166	4.903	0.02
Within all samples	0.278	1591.921	72.158	0.01

However, nuclear SNPs revealed a putative hybrid zone between Northern and Southern populations, occurring at the switch‐point region between mt‐haplogroups (between Mackay and Townsville in the Burdekin Gap). Our DAPC analyses revealed a best fit for data with 3–5 groups (*K* = 3, *K* = 4, *K* = 5; Figure S6) in which one group in each case was a ‘north–south hybrid zone’ population (comprising individuals from Townsville, Pentland, Bowen, and Mackay), distinct from both Northern and Southern populations (Figure 2C **)**. Moreover, simulated F1 and F2 north–south hybrids (synthesised using parent genotypes from areas further north and south) were assigned to the same group as the ‘north–south hybrid zone’ (Figure 2C).

Brisbane, at the southern range edge, represented a second ‘hybrid zone’ between Northern and Southern populations. All mito‐NQ samples (*N* = 8) from Brisbane grouped with the Brisbane mito‐SQ individuals in the PCA analysis (Figure 2B) and more broadly with the Southern population in DAPC analysis (Figure 2C), consistent with interbreeding between the haplogroups in Brisbane. Furthermore, the Brisbane population was more similar to that of north–central Queensland than would be expected from geography alone (Figure 2B).

Surprisingly, despite their divergent haplotype (mito‐CY), our Cape York samples clustered with the Northern population in our DAPC analyses, unless we allowed for values of *K* = 5 (Figure 2C). Thus, it seems likely that some nuclear gene flow is also occurring between Cape York and Northern populations. Also unexpectedly, *T. hockingsi* from one lowland coastal region of the Northern population (Cairns, *N* = 8) differed substantially at nuclear SNPs from those sampled elsewhere throughout the north, despite sharing the mito‐NQ haplotype (Figure 2A–C; Figure S4).

### 3.4 Nuclear Loci That Contribute to Population Divergence

Using our DArTseq SNP dataset, we identified 83 outlier SNPs (top 1% of *F*_*ST*_ values) that differentiated all ‘non‐hybrid’ mito‐NQ versus mito‐SQ individuals, and 83 outlier SNPs that differentiated the subset of ‘non‐hybrid’ mito‐NQ and mito‐SQ adjacent to the Burdekin Gap hybrid zone, with 34 SNPs common to both comparisons. We also identified 35 loci via DAPC analysis (in other words, SNPs disproportionately involved in clustering of the discriminant between the Northern and southern populations). In total, 69 loci were identified by the DAPC and *F*_*ST*_ methods, 15 of which were identified by both the *F*_*ST*_ and DAPC analyses (Table S4).

We identified 77 genes within 5000 bp of these 69 SNP loci. Of these, 10 genes (13%) were predicted by MULocDeep to produce proteins that are transported to the mitochondria (Tables S5–S7). Using BLAST, 48 genes (62%) could be identified, each of which were associated with one or more GO terms, which define their functional characteristics. This included three genes that could be categorised as N‐mt genes based on GO terms: that is, they encoded proteins that were used within the mitochondria (all three were also identified by MULocDeep). These genes had various roles in the mitochondria, including the pseudouridylation of mitogenome‐encoded mRNAs (TRUB2; (Antonicka et al. 2017; Arroyo et al. 2016)), mitochondrial autophagy (BNIP‐3; (Liu et al. 2022; Yasuda et al. 1998)) and ferroptosis, a kind of programmed cell death (ACSF2; (Dixon et al. 2012)). Other genes with identified homologues include those related to cell death and apoptosis, and one odour receptor (Tables S5–S7).

## 4 Discussion

Previous evidence has suggested divergent mitochondrial lineages exist within the Australian stingless bee *T. hockingsi* (Brito et al. 2014; Franck et al. 2004; Françoso et al. 2019). Here we confirm that this species is characterised by three mitochondrial haplogroups that correspond to discrete geographic regions in eastern Australia: Southern Queensland, Northern Queensland, and Cape York. The sequence divergence between these haplogroups is high (12%–14% pairwise nucleotide difference), similar to that observed between genera for most stingless bees (average: 13%; Françoso et al. 2019) and approaching that observed between *T. hockingsi* and its near relative *T. carbonaria* (16% at mt‐COI; Françoso et al. 2019). Moreover, sequence divergence between *T. hockingsi*'s three mitochondrial haplogroups includes changes in amino acid sequence at all 13 protein‐coding genes. We found that nuclear markers similarly indicated strong regional genetic differentiation in *T. hockingsi*, broadly consistent with the three mitochondrial haplogroups. Yet gene flow is occurring between haplogroup‐defined populations in at least two regions: at the contact point of Northern and Southern populations in central Queensland, and in a southern region (Brisbane) where beekeeping has brought together the previously isolated mitochondrial lineages. Thus, reproductive isolation between *T. hockingsi*'s populations is incomplete.

*Tetragonula hockingsi*'s population genetic structure broadly aligns with the biogeographic features of north‐eastern Australia (Bryant and Krosch 2016; Ebach et al. 2013) and the effects of strong male‐biased dispersal (Bueno et al. 2022), which in combination can produce discordance between mitochondrial and nuclear DNA (Toews and Brelsford 2012). Southern, Northern, and Cape York populations each occupy largely forested biomes (Cracraft 1991; Ebach et al. 2013), separated from each other by stretches of drier, more open woodland or savannah (the Burdekin Gap and Laura Basin/Black Mountain Corridor respectively; Bryant and Krosch 2016). These drier, less‐forested regions likely arose during Pleistocene climate changes (2.58 mya to 11,700 ya); (Bryant and Krosch 2016), and have been implicated in the vicariance or speciation of a variety of eastern Australian forest taxa, including mammals, reptiles, and insects (Bryant and Fuller 2014; Chapple et al. 2011; Watson and Theischinger 1984). For stingless bees, areas of low tree density likely correspond to areas of low colony density, because trees provide both floral sources and nest sites. Importantly; however, these less‐forested regions will limit female dispersal significantly more than male dispersal, generating greater structuring in mitochondrial than nuclear genomes. This is because stingless bee queens can disperse only into colonies prepared and provisioned by workers, with workers transporting food and building materials from the parent nest to a new nest site over a period of many months (Grüter 2020). New colonies must therefore be established within the worker flight range of the parent colony, a distance of around 700 m in *Tetragonula* (Smith et al., 2017). In contrast, male stingless bees are highly capable dispersers that regularly travel several kilometres from their natal nests, and as much as 20 km, before mating (Bueno et al. 2022; dos Santos, Imperatriz‐Fonseca, and Arias 2016). Thus, male dispersal likely accounts for most of the gene flow between populations of *T. hockingsi* (excluding that arising from recent human‐mediated dispersal), with males capable of traversing unforested areas that females cannot. Similar mito‐nuclear discordance has also been observed in other stingless bees for instance, *Partamona helleri* in Brazil (Dessi et al. 2022).

Geographic barriers can also explain some of the finer‐scale population structuring observed within the Northern *T. hockingsi* population. In Far North Queensland, *T. hockingsi*'s range spans both the low altitude, coastal region of Cairns and the higher altitude regions of the Atherton Tablelands (750 m a.s.l; part of Australia's Great Dividing Range; (Nix 1991; Ollier 1982)). Nuclear markers indicated that Cairns *T. hockingsi* are distinct from those elsewhere in the north, a genetic divergence that might reflect adaptation to the distinct climate of the coast (warmer, with higher humidity) relative to the uplands (cooler, with higher rainfall), or may be due simply to the partial isolation created by the range. Interestingly, Cairns *T. hockingsi* shared the same mitochondrial haplogroup with others in the Northern population, so nuclear divergence in this case is somewhat greater than we would expect, relative to mitochondrial divergence.

Beekeeping has affected the distribution and population genetic structure of many social bees across the world in the past century, including various species of honey bee, bumble bee, and stingless bee (Byatt et al. 2016; Chahbar et al. 2013; Chapman et al. 2017; Francisco et al. 2014; Jaffe et al. 2016; Jensen et al. 2005; Rangel et al. 2016). *Tetragonula hockingsi* represents a valuable system in which to examine the potential effects of such movements in bees, given that hive trade of this species is becoming increasingly common (Halcroft et al. 2013). On the one hand, hive movements by humans might increase local genetic diversity and facilitate the adaptation of bee populations to environmental change (Chapman et al. 2017; Todesco et al. 2016). Conversely, hive trade might increase the incidence of low fitness interpopulation hybrids if genetic incompatibilities and reproductive interference occur, or lead to the loss of locally adapted alleles (Byatt et al. 2016; Todesco et al. 2016). Human activity has clearly already impacted *T. hockingsi*'s population genetics in at least the southern part of its range, though the phenotypic outcomes of this admixture remain unknown. Brisbane has been a hub of stingless beekeeping in Australia for several decades, and the genetic signature of Brisbane's *T. hockingsi* today reflects an admixture of diverse sources from across Queensland, including Northern, Southern and even Cape York types (based on the presence of 1.3% mito‐CY haplotypes). Mito‐NQ and mito‐CY males also joined the mating aggregations that formed at colonies with mito‐SQ queens. In stingless bees, the presence of males in mating aggregations does not necessarily equate to short‐range mate attraction and hybridization; for instance, some *T. carbonaria* males will join *T. hockingsi* aggregations, despite showing no attraction to *T. hockingsi* queens at close range (Paul et al. 2023). Moreover, Northern and Southern *T. hockingsi* populations have presumably had little opportunity to evolve pre‐zygotic barriers to mating in response to genetic incompatibilities. Nevertheless, the male aggregation behaviour of *T. hockingsi* with imported haplotypes in Brisbane was consistent with the genetic evidence for admixture in this region.

Whether hive movements by beekeepers have also influenced the population genetics of the central Queensland region between Northern and Southern populations is less clear. We consider this region to most likely represent a natural hybrid zone, in which the two populations are largely isolated but occasional male dispersal across the Burdekin Gap maintains some gene flow. This is because we sampled very few ‘misplaced’ haplotypes from hived colonies on either side of the divide (just a single mito‐SQ from Townsville, and a single mito‐NQ from Mackay from 98 total hives sampled in this ‘hybrid region’). However, we cannot rule out that the population genetic pattern we observe has instead arisen from the human‐aided dispersal of hives between Townsville and Mackay in recent decades. In this case, our failure to sample misplaced haplotypes in the region could reflect a sampling‐bias towards beekeepers who only source hives locally, and/or there may be strong selection for local mitochondrial haplotypes in this region (Dowling, Friberg, and Lindell 2008), such that colonies translocated over the gap are frequently usurped by those with local female lineages (Gloag et al. 2008).

Mito‐nuclear coevolution theory predicts that where isolated populations have highly divergent mitogenomes, interpopulation hybrids are likely to experience genetic incompatibilities (mito‐nuclear BDMIs) and that these may promote reproductive isolation (Burton and Barreto 2012; Ellison and Burton 2008; Hill 2016). This is because nuclear genes whose products interact closely with mitochondrial genes (N‐mt genes) should evolve in population‐specific ways to compensate for any changes in mt‐genes and thereby maintain optimal respiration (Havird and Sloan 2016; Lechuga‐Vieco, Justo‐Méndez, and Enríquez 2021). Ultimately, whole genome data is needed to assess the extent to which N‐mt genes have diverged via selection (in other words, co‐adaptated with mitogenomes) between *T. hockingsi* populations. However, we see some support for the predictions of mito‐nuclear coevolution from our SNP dataset. Among the most highly differentiated loci between Northern and Southern populations, 13% were associated with genes predicted to localise in mitochondria, and three of those genes were homologues of known N‐mt genes. For comparison, using the curated GLAD database (Hu et al. 2015) and ncbiRefSeq annotated nuclear genes in the *Drosophila melanogaster* dm6 genome (August 2014 release, accessed using https://genome.ucsc.edu/), we can estimate that approximately 2.84% of *D. melanogaster* nuclear genes are N‐mt genes. This coarse approximation suggests that N‐mt genes may be over‐represented among the set of candidate genes causing BDMIs between *T. hockingsi* populations, relative to the expected number of N‐mt genes in the nuclear genome.

One N‐mt gene that we identified was *TRUB2*, which is involved in modifying mitogenome‐encoded mRNAs via the addition of pseudouridine: a lack of pseudouridylation performed by *TRUB2* can lead to decreased translation and functional deficiencies in mt rRNAs, with serious implications for respiration (Antonicka et al. 2017; Arroyo et al. 2016). This and other associations between outlier SNPs and N‐mt genes that we observe in *T. hockingsi* are therefore consistent with selection on different N‐mt alleles in Northern and Southern populations. Other outlier SNPs were linked with genes that had no evident direct association to mitochondrial gene products, but which might be involved in nuclear‐nuclear BDMIs between populations, including genes related to cell death and apoptosis, where mismatches are likely to have strong effects on fitness (Lane 2011; Zhang, Montooth, and Calvi 2017), and an odour‐receptor gene, where odour plays a key role in insect mate recognition (Crowley‐Gall et al. 2016; Smadja and Butlin 2009).

Assuming that mito‐nuclear coevolution has occurred between at least some of the mt‐genes and N‐mt genes of each of *T. hockingsi*'s populations, the observed patterns of inter‐population gene flow align closely with the expectations of mito‐nuclear incompatibility between diverged populations. Under this scenario, mito‐nuclear BDMIs would reduce the average fitness of inter‐population hybrids in *T. hockingsi* (Hill 2019; Sloan, Havird, and Sharbrough 2017) but gene flow still persists at nuclear loci other than N‐mt genes (Cairns et al. 2021). That is, there is co‐introgression of both mt and N‐mt genes in hybrids, such that we observe a sharp transition in these genes over geographic space, yet gradual transition for other nuclear genes (Hill 2019). Over time, such mito‐nuclear BDMIs may serve to promote reproductive isolation and speciation because they favour secondary barriers to evolve.

However, this outcome will depend on the extent to which hybrid fitness is affected. Cases of mito‐nuclear discordance are not uncommon and can be produced by a variety of demographic processes (Toews and Brelsford 2012). For instance, the butterfly *Thymelicus sylvestris* (Poda 1761) has several sympatric diverged mitochondrial lineages that are incongruent with nuclear lineages, as measured using whole‐genome SNPs, and reveal extensive gene flow occurring despite mitochondrial divergence (Hinojosa et al. 2019). This and other reported cases of mitochondrial introgression can be interpreted as evidence that mito‐nuclear incompatibilities are often not strong enough to promote speciation events (Angers et al. 2018; Burton 2022; Makhov, Gorodilova, and Lukhtanov 2021; Qi et al. 2014; Sloan, Havird, and Sharbrough 2017). The evolutionary outcome of mito‐nuclear BDMIs may also be influenced by maternally transmitted endosymbionts, such as *Wolbachia*, that promote their own transmission and any associated mitochondrial haplotypes (Jiggins 2003). Studies to date of the microbiota of Australian *Tetragonula* have not detected *Wolbachia* (Hall et al. 2020; Leonhardt and Kaltenpoth 2014; Liu et al. 2023; Mills et al. 2023), though this endosymbiont has been reported in some Afrotropical stingless bees (Tola et al. 2021) and more broadly within Apidae (Gerth, Geissler, and Bleidorn 2011).

Importantly, the negative effect of BDMIs on fitness may manifest more acutely in some environmental conditions than others (Sloan, Havird, and Sharbrough 2017; Tobler, Barts, and Greenway 2019). Gene × gene × environment (G × G × E) impacts of temperature and other environmental factors on mito‐nuclear incompatibilities have been demonstrated in several laboratory‐based studies (Hoekstra, Siddiq, and Montooth 2013; Rand et al. 2021; Tobler, Barts, and Greenway 2019). These temperature‐dependent effects can occur, for instance, because mt and N‐mt proteins are more disrupted at higher temperatures or because energy use is higher in warmer conditions and thus the demand on the mitochondria to produce energy is greater (Hoekstra, Siddiq, and Montooth 2013). Thus, the fitness of north–south *T. hockingsi* hybrids in Brisbane may be quite different to those from the Burdekin Gap, where climates are hotter and drier. Further *in silico* analysis comparing samples from both hybrid regions will help to reveal any such G × G × E mito‐nuclear effects.

The sequence of population changes that ultimately leads to the formation of new species is not always linear and may involve the repeated merger and divergence of populations over time. *Tetragonula hockingsi* populations appear to exist currently in the grey zone of this two‐way ‘speciation continuum’ (Turelli, Barton, and Coyne 2001) despite clear mitochondrial and nuclear structuring. Whether the different populations of *T. hockingsi* continue on trajectories towards greater differentiation or greater gene flow depends on the strength and existence of barrier loci (including mito‐nuclear loci) that have developed between the populations (Angers et al. 2018; Despres 2019; Hinojosa et al. 2019). The impact of human activities on these trajectories is also uncertain. Both direct impacts from beekeeping activities and indirect impacts from changing climates and land use are likely to contribute to *T. hockingsi*'s future population structure, and the species' resilience. Future work could aim to better characterise the divergence in nuclear genomes between *T. hockingsi*'s three populations, and to investigate more directly the fitness of inter‐population hybrids via further sampling in both the natural and artificial hybrid zones. In particular, we propose that *T. hockingsi* is a highly tractable model for future research on mito‐nuclear coevolution in natural populations, and the role of mito‐nuclear incompatibilities in population divergence and speciation.

## Author Contributions

**Genevieve Law:** conceptualization (equal), data curation (equal), formal analysis (equal), investigation (equal), methodology (equal), resources (equal), visualization (equal), writing – original draft (equal), writing – review and editing (equal). **Carmen R. B. da Silva:** resources (supporting), writing – review and editing (equal). **Inez Vlasich‐Brennan:** resources (supporting), writing – review and editing (equal). **Benjamin A. Taylor:** resources (supporting), writing – review and editing (equal). **Brock A. Harpur:** resources (supporting), writing – review and editing (equal). **Tim Heard:** resources (supporting), writing – review and editing (equal). **Scott Nacko:** resources (supporting), writing – review and editing (equal). **Markus Riegler:** resources (supporting), writing – review and editing (equal). **James B. Dorey:** resources (supporting), writing – review and editing (equal). **Mark I. Stevens:** resources (supporting), writing – review and editing (equal). **Nathan Lo:** conceptualization (equal), methodology (equal), supervision (supporting), writing – original draft (supporting), writing – review and editing (equal). **Rosalyn Gloag:** conceptualization (lead), data curation (equal), funding acquisition (lead), investigation (equal), methodology (equal), project administration (lead), resources (equal), supervision (lead), visualization (supporting), writing – original draft (equal), writing – review and editing (equal).

## Conflicts of Interest

The authors declare no conflicts of interest.

## Data Availability Statement

The sequence data that support the findings of this study are openly available in NCBI's Sequence Read Archive (Accession Numbers SAMN42501115–SAMN42501231; BioProject: PRJNA1136031) and Genbank (Accession Numbers PQ058352–PQ058454 and PQ163738–PQ163743). All other data that supports the findings of this study are available in the Supporting Information of this article.

## Supporting information


Appendix S1.

Appendix S2.
