# Supplementary material for: Gene Flow Between Populations With Highly Divergent Mitogenomes in the Australian Stingless Bee, Tetragonula hockingsi
Source: Ecol Evol. 2024 Nov 13;14(11):e70475. doi: 10.1002/ece3.70475 (PMC11560288; doi:10.1002/ece3.70475)
Supplement: Supplementary file 1 — Appendix S1. [file ECE3-14-e70475-s001.docx]

**Supplementary Information**

**Gene flow between populations with highly divergent mitogenomes in the Australian stingless bee, *Tetragonula hockingsi***

Law G, Da Silva C, Vlasich-Brennan I, Taylor B, Harpur B, Heard T, Nacko S, Riegler M, Dorey J, Lo N, & Gloag R.

Supplementary Information in this file:

**Supplementary Methods**. Confirmation of sex for individuals from mating aggregations

**Table S2.** Primers used in PCR amplification of *COI* sequences and microsatellites.

**Table S3.** Substitution models for mt-gene tree of *T. hockingsi* haplogroups.

**Figure S1.** An ML phylogenetic tree of *T. hockingsi* constructed using *COI* sequences.

**Figure S2**. Locations in Brisbane, Queensland where *T. hockingsi* workers were collected.

**Figure S3.** Locations in Brisbane, Queensland where *T. hockingsi* male mating aggregations were sampled and the proportion haplogroup in each aggregation.

**Figure S4.** ML phylogenetic tree plotted using nuclear SNPs from 90 *T. hockingsi*.

**Figure S5.** Heatmap of pairwise F_ST_ values between *T. hockingsi* from each sampling region.

**Figure S6.** Bayesian information criterion (BIC) values for each number of clusters (K) generated as a part of DAPC to assess population structure in *T. hockingsi* nuclear SNPs.

Supplementary Information in the file “Supplementary Tables.xlsx”:

**Table S1.** Collection, species, and haplotype information for samples used in all analyses.

**Table S4**. Outlier SNP loci used to assess divergence in N-mt genes between populations.

**Table S5**. Genes located within 5000 bp of outlier SNPs and protein localization predictions.

**Table S6**. Primary gene ontology (GO) terms for genes linked with outlier SNP loci.

**Table S7**. Function of N-mt genes linked with outlier SNP loci.

***Supplementary methods:***

**Confirmation of sex for individuals from male mating aggregations**

Male and female *Tetragonula* spp. are similar in size and morphology (Dollin et al., 1997). Following placement into ethanol, individuals can be sexed either by inspection under a dissecting microscope or by assessing ploidy level at molecular markers. To confirm that all individuals collected from mating aggregations were actually males and not by-catch of workers from nearby colonies, we first genotyped all putative male samples (N = 813), plus 96 known workers from Brisbane for comparison, at five microsatellite loci (Tc3.155, Tc3.302. Tc4.214, Tc4.287 and Tc4.63; see Table A2.1) (Green et al., 2001). Male bees are haploid and females are diploid; thus, males are expected to be hemizygous at all loci, while individuals that were heterozygous at one or more loci must be female.

We amplified samples in reaction volumes of 5 μL: 1.75 μL of Milli-Q water, 0.25 μL of glycerol, 0.5 μL of PCR buffer, 0.4 μL of 25 mM MgCl2, 0.5 μL of 2 mM dNTPs, 0.04 μL of Taq-Ti, and primers (see Table A2.1). We used the following PCR conditions: 94 C for 8 minutes, followed by 29 cycles of denaturation (94 C for 30 seconds), annealing (56 C for 30 seconds) and extension (72 C for 30 seconds) (Green et al., 2001; Paul et al., 2023). PCR products were analysed on a 3130x Genetic Analyser (Life Technologies, USA) and genotypes scored using GeneMapper (Applied Biosystems, USA).

In total, 30 females were removed from the male aggregation dataset as they were heterozygous at one or more loci and subsequently confirmed via morphology to be workers (Bueno et al., 2022). A further 45 samples for which no genotype was returned at most or all microsatellite loci were also sexed morphologically and determined to be males. As some known females (10.42%) also returned homozygous genotypes at all five loci, some putative males that were homozygous for all loci may therefore have been incorrectly identified as males. However, a random sample of 21 males that were homozygous for all loci were morphologically checked and found to be males.

**Table S2.** Primers used in PCR amplification of *COI* sequences (Barhock and Folmer primers) and microsatellites (Tc primers) for *Tetragonula hockingsi* and other *Tetragonula* species.

| **Primer** | **Direction** | **Primer sequence** | **Source** |
| --- | --- | --- | --- |
| BarhockF | Forward | CTCCATTGTTACTGGGCATGC | (Françoso et al., 2019) |
| BarhockR | Reverse | AAGGCCGAATCCTGGAAGAA | (Françoso et al., 2019) |
| LCO1490 | Forward | GGTCAACAAATCATAAAGATATTGG | (Folmer et al., 1994) |
| HCO2198 | Reverse | TAAACTTCAGGGTGACCAAAAAATCA | (Folmer et al., 1994) |
| Tc.3 155 | Forward | AGAATCACGTCGGCATCCGGA | (Green et al., 2001) |
| Tc.3 155 | Reverse | CTTGAAATCCAGCGCAGAGTG | (Green et al., 2001) |
| Tc.3 302 | Forward | CGATTTTACGGGCATCCTTCG | (Green et al., 2001) |
| Tc.3 302 | Reverse | CATCGTTAGGGACACGTGCAA | (Green et al., 2001) |
| Tc.4 214 | Forward | CGCAGTATGCAGTTAAAGAATC | (Green et al., 2001) |
| Tc.4 214 | Reverse | GTAATCTTGTAATTGACTAACTCG | (Green et al., 2001) |
| Tc.4 287 | Forward | TCCACCGCGATACGATGGTAC | (Green et al., 2001) |
| Tc.4 287 | Reverse | GTAATACAACGCGGCTTCCTC | (Green et al., 2001) |
| Tc4. 63 | Forward | GAGATGGTGTGGGACAACGTG | (Green et al., 2001) |
| Tc4. 63 | Reverse | TGACGAGACGCCTGACAGAAC | (Green et al., 2001) |

**Table S3.** Appropriate substitution models identified using IQ-TREE’s ModelFinder (Kalyaanamoorthy et al., 2017) in relation to an ML phylogeny of concatenated amino acid sequences translated from mitochondrial genes of *Tetragonula* species and *T. hockingsi* haplogroups (see Figure 1).

| **Gene name** | **Model** |
| --- | --- |
| *ATP6* | mtMet+G4 |
| *ATP8* | mtMet+G4 |
| *CO2* | mtMet+G4 |
| *CO3* | mtMet+G4 |
| *COI* | mtART+I |
| *CytB* | mtMet+G4 |
| *NAD1* | mtInv+G4 |
| *NAD3* | mtMet+G4 |
| *NAD4* | mtMet+G4 |
| *NAD5* | mtVer+F+G4 |
| *NAD6* | mtMet+G4 |
| *ND2* | mtMet+G4 |
| *ND4L* | mtInv+I |

******

**Figure S1.** An ML phylogenetic tree of *T. hockingsi* constructed using *COI* sequences, to assess the origin of mito-NQ and mito-SQ haplotypes in Brisbane. This tree includes all samples from Brisbane, plus samples from across the species’ distribution (see Table S1). The tree was constructed using IQ-TREE and plotted using ape. The scale on the x axis represents genetic distance. Node support values are bootstrap values calculated with UFBoot2 in IQ-TREE. Circles indicate haplogroup (mito-CY, mito-NQ, or mito-SQ) and sample name text colour indicates the broad location of the sample (Cairns, Atherton Tablelands, Northern–Southern hybrid zone, Southern population). The tree was rooted using three *T. carbonaria COI* sequences from Brisbane (OO069, OO036, and CH001; see Table S1).

**Figure S2**. Locations in Brisbane where *T. hockingsi* colonies (or workers from flowers) were sampled and whether they were mito-SQ (light green) or mito-NQ (dark green) haplotypes at mt-*COI*. Rescued colonies (i.e. those removed recently from natural locations and installed in hives) were plotted as the location at which they were acquired. Rescued colonies whose original location had been recorded only as “Brisbane” or “Northside” (meaning north of the Brisbane River) are not plotted here (n = 96). Similarly, the two colonies with the mito-CY haplotype were not plotted, as their exact original location was not known.

**Figure S3.** **A**. Locations in Brisbane where *T. hockingsi* male mating aggregations (n = 15) were sampled. Sampling location is marked with a coloured point to represent the haplotype of the queen (inferred from her workers) attracting the aggregation. At eleven locations, only mito-SQ queens were present. At four locations, the aggregation was equidistant to two or more colonies of different haplotypes (mito-SQ and mito-NQ *T. hockingsi*) or species (mito-SQ *T. hockingsi* and *T. carbonaria*). Map created using leaflet in R. **B**. The proportion of males of each *T. hockingsi* haplotype (mito-CY, mito-NQ, mito-SQ) and species (*T. carbonaria*) found in fifteen male mating aggregations, and the number of males haplotyped per aggregation. Aggregation numbers (1–15) correspond to the sampling locations in A.

**Figure S4.** ML phylogenetic tree plotted using nuclear SNPs from *Tetragonula hockingsi* sampled from across the species’ range (n=90), as well as outgroup species: *T. clypearis* (n=2), *T. sapiens* (n=1) and *T. carbonaria* (n=1). Sample names are coloured according to geographic location (Cape York, Cairns, Atherton Tablelands, Northern-Southern hybrid zone, Southern Queensland), while circles are coloured according to the sample’s mitochondrial haplogroup. The tree was plotted using IQ-TREE and reformatted using ape in R. Bootstrap values are displayed at nodes and were determined using UFBoot2.


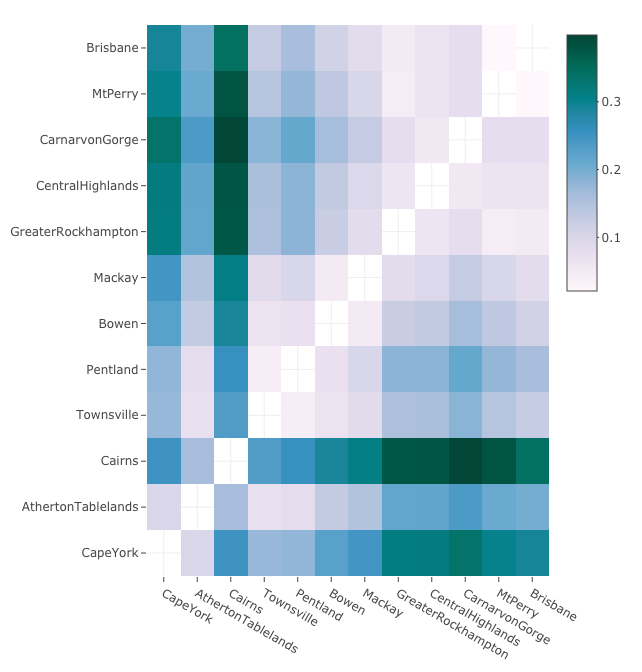


**Figure S5.** Heatmap of pairwise F_ST_ values between each region (subpopulation) of *T. hockingsi* sampled in this study. Cooktown and Maryborough are excluded because only one individual was collected from each of these populations. Pairwise F_ST_ values calculated using StAMPP.


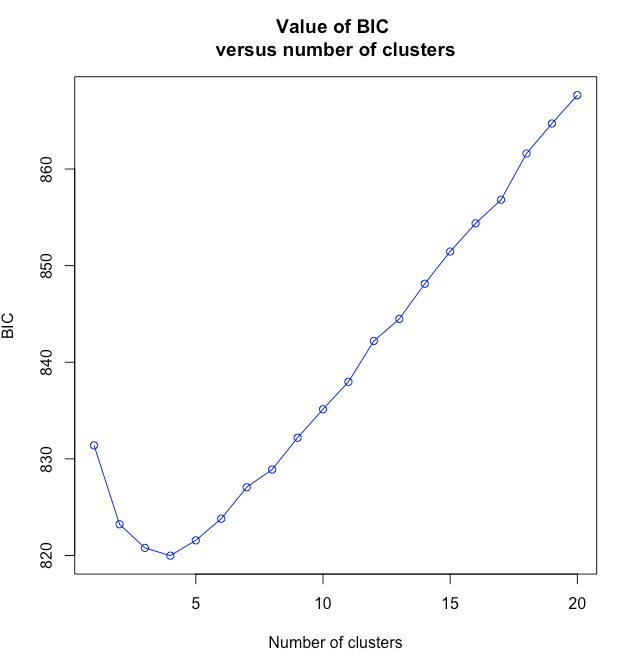


**Figure S6.** Bayesian information criterion (BIC) values representing support for each number of clusters (K) generated as a part of DAPC using adegenet in R. DAPC analysis was conducted on nuclear SNPs of 90 samples of *T. hockingsi*, as well as simulated hybrid individuals (see Figure 2, Table S1).

**Reference List**

Bueno, F. G. B., Bueno, B. G. B., Buchmann, G., Heard, T., Latty, T., Oldroyd, B. P., Hosoi, A. E., & Gloag, R. (2022). Males Are Capable of Long-Distance Dispersal in a Social Bee. *Frontiers in Ecology and Evolution*, *10*. <https://doi.org/10.3389/fevo.2022.843156>

Dollin, A. E., Dollin, L. J., & Sakagami, S. F. (1997). Australian stingless bees of the genus Trigona (Hymenoptera : Apidae). *Invertebrate Taxonomy*, *11*(6), 861-896. <https://doi.org/10.1071/it96020>

Folmer, O., Black, M., Hoeh, W., Lutz, R., & Vrijenhoek, R. (1994). DNA primers for amplification of mitochondrial cytochrome c oxidase subunit I from diverse metazoan invertebrates. *Mol Mar Biol Biotechnol*, *3*(5), 294-299.

Françoso, E., Zuntini, A. R., Ricardo, P. C., Silva, J. P. N., Brito, R., Oldroyd, B. P., & Arias, M. C. (2019). Conserved numts mask a highly divergent mitochondrial-COI gene in a species complex of Australian stingless bees Tetragonula (Hymenoptera: Apidae). *Mitochondrial DNA Part A*, *30*(7), 806-817. <https://doi.org/10.1080/24701394.2019.1665036>

Green, C. L., Franck, P., & Oldroyd, B. P. (2001). Characterization of microsatellite loci for Trigona carbonaria, a stingless bee endemic to Australia. *Molecular Ecology Notes*, *1*(1-2), 89-92. <https://doi.org/https://doi.org/10.1046/j.1471-8278.2001.00041.x>

Kalyaanamoorthy, S., Minh, B. Q., Wong, T. K. F., von Haeseler, A., & Jermiin, L. S. (2017). ModelFinder: fast model selection for accurate phylogenetic estimates. *Nature Methods*, *14*(6), 587-589. <https://doi.org/10.1038/nmeth.4285>

Paul, G., Bartels, L., Bueno, F. G. B., Law, G., Heard, T., Chapman, N., Buchmann, G., Lim, J., & Gloag, R. (2023). Shifting range in a stingless bee leads to pre-mating reproductive interference between species. *Conservation Genetics*, *24*(4), 449-459. <https://doi.org/10.1007/s10592-023-01512-7>
